# Psychosocial Interventions for Patients Undergoing Gastrointestinal Cancer Surgery: A Systematic Review

**DOI:** 10.1245/s10434-026-19788-7

**Published:** 2026-05-25

**Authors:** Olivia Monton, Kimberly Kopecky, Ann-Margret Ervin, Shannon Fuller, Lori Rosman, Michael J. Marcaccio, Fabian M. Johnston

**Affiliations:** 1 Department of Surgery, McMaster University, Hamilton, ON Canada; 2 Department of Epidemiology, Johns Hopkins Bloomberg School of Public Health, Baltimore, MD USA; 3 Division of Surgical Oncology, Department of Surgery, University of Alabama at Birmingham Heersink School of Medicine, Birmingham, AL USA; 4 Department of Health, Behavior and Society, Johns Hopkins Bloomberg School of Public Health, Baltimore, MD USA; 5 Johns Hopkins University Welch Medical Library, Baltimore, MD USA; 6 Department of Surgery, Wake Forest University School of Medicine, Winston-Salem, NC USA

**Keywords:** Psychosocial interventions, Psychosocial oncology, Psychological, Interventions, Gastrointestinal cancer surgery, GI cancer surgery

## Abstract

**Background:**

Psychosocial interventions may address the psychosocial challenges associated with a cancer diagnosis and treatment. Limited research exists on their effectiveness for patients undergoing gastrointestinal cancer surgery. The objective of this study was to summarize existing evidence on psychosocial interventions for patients with gastrointestinal cancer undergoing surgery by characterizing interventions and assessing their impact on quality of life, anxiety, and depression.

**Materials and Methods:**

We searched five electronic databases on 1 December 2023 (updated 20 December 2024) to identify randomized controlled trials evaluating psychosocial interventions in adult patients with gastrointestinal cancer undergoing surgical treatment. We performed title and abstract screening and full text review, followed by data abstraction and narrative synthesis to describe intervention characteristics and their impact on quality of life, anxiety, and depression.

**Results:**

A total of 27 studies published between 2005 and 2024 were included. Interventions varied by therapeutic modality and included education (*n* = 4, 14.8%), behavioral training (*n* = 4, 14.8%), psychotherapy (*n* = 4, 14.8%), emotional support (*n* = 2, 7.4%), education and emotional support (*n* = 9, 33.3%), and other multimodal strategies (*n* = 4, 14.8%). Among the studies that assessed impact on quality of life, anxiety, and/or depression, significant improvements were reported in quality of life (9/16, 56.3%), anxiety (12/16, 75%), and depression (8/12, 66.7%).

**Conclusions:**

This review provides evidence that psychosocial interventions may improve quality of life, anxiety, and depression in patients with gastrointestinal cancer undergoing surgery. However, the interventions varied widely by therapeutic modality, delivery setting, mode of delivery, timing, and duration. Further research is needed to identify the most effective intervention components and target subpopulations.

**Supplementary Information:**

The online version contains supplementary material available at 10.1245/s10434-026-19788-7.

The burden of gastrointestinal (GI) cancer remains high, with more than 5 million new cases and more than 3.6 million deaths reported worldwide in 2020.^1^ Surgical resection is a common treatment for many GI cancers but carries higher postoperative risk than surgery for benign diseases, including longer hospital stays, increased complication rates, and higher mortality.^2^

While perioperative optimization programs have traditionally focused on addressing biomedical risk factors,^3,4^ psychosocial factors such as anxiety, depression, social support, and coping skills are increasingly recognized as important determinants of postoperative outcomes in patients with GI cancer.^5^ In a cohort study of patients undergoing GI cancer surgery, medically complex patients with multiple psychosocial risk factors had 3.37-fold odds of developing a postoperative complication compared with patients with no or 1 psychosocial risk factor.^5^ Notably, nearly 75% of patients in this study reported at least 1 psychosocial risk factor.

Patients with GI cancer may be particularly vulnerable to psychosocial distress due to a high symptom burden (e.g., pain, fatigue, loss of appetite, weight loss) and the complexity of treatment.^6,7^ These challenges may be further compounded by surgical interventions, which can result in long-term sequelae (e.g., stoma creation and management) as well as functional impairments (e.g., altered bowel function) that can be stigmatizing and isolating.^8–10^ Unfortunately, psychosocial interventions remain underutilized and understudied in this population. A 2014 systematic review assessing the efficacy of psychosocial interventions on patients with GI cancer found that only two of eight randomized controlled trials (RCTs) were conducted in surgical patients.^11^ To our knowledge, no systematic reviews have assessed the effectiveness of psychosocial interventions in patients undergoing GI cancer surgery and our study aimed to fill this gap in the literature. The two specific aims of this review were: (1) to characterize psychosocial interventions and (2) to assess their impact on quality of life, anxiety, and depression in patients with GI cancer undergoing surgery.

## Materials and Methods

This review was conducted using Cochrane Intervention Review standards (C1-C75),^12^ and reported following the Preferred Reporting Items for Systematic Reviews (PRISMA) guidelines.^13^ Institutional Review Board approval was not required.

### Study Outcomes

The primary outcome was quality of life, and the secondary outcomes were anxiety and depression, with no restrictions on the types of measurement instruments or the timing of outcome assessments.

### Eligibility Criteria

We included all RCTs published in English that evaluated the effectiveness of a psychosocial intervention in patients with GI cancer undergoing surgery that met the following inclusion criteria: (1) study design was a RCT where participants were assigned to one or more psychosocial intervention versus standard of care; (2) study population included adult patients with GI cancer (esophagus, stomach, small intestine, pancreas, liver, gallbladder, colon, rectum, anus) undergoing elective surgical treatment; and (3) study evaluated the impact of a psychosocial intervention delivered before and/or after surgery. There were no date restrictions.

We excluded primary studies that were not RCTs (e.g., cohort studies, case-control studies, and non-randomized trials), secondary literature (e.g., review articles), unpublished research (e.g., conference abstracts), and ongoing research (e.g., study protocols and clinical trial registries). However, abstracts, protocols, and trial registries were used to identify peer-reviewed publications that met our inclusion criteria. Studies evaluating physical or nutritional interventions in isolation were excluded. Multimodal prehabilitation or rehabilitation programs that involved a psychosocial component were only included if they directly measured the effect of the psychosocial component in isolation. Furthermore, studies that evaluated psychosocial interventions, which addressed specific consequences of surgical treatment, such as low anterior resection syndrome, were excluded.

### Search Strategy

Our search strategy (outlined in Table A1, Supplementary Material) was developed in collaboration with an informationist at the Johns Hopkins Welch Medical Library. We searched five electronic databases (PubMed, Embase, Cochrane Trials, CINAHL, and PsycInfo) on 1 December 2023, using a combination of controlled vocabulary and keyword terms. Our search strategy included three concepts: gastrointestinal cancer, oncologic surgical resection, and psychosocial intervention. We performed citation searching of included studies and review articles to identify all relevant articles. An updated search using the original search strategy was performed on 20 December 2024 to capture additional articles published after our initial search.

### Study Selection

Search results were imported into Covidence (Veritas Health Innovation, Australia).^14^ Duplicates were removed by Covidence and manually by two reviewers (OM, KK). Prior to manual screening, we applied the Covidence automation tool, which leverages machine learning and automation,^15–17^ to remove references not reporting on RCTs. Given the novelty of this tool, we reviewed 10% of the ineligible studies for accuracy. Duplicates identified manually by the reviewers were not included in the 10% sample of references classified as ineligible by the automation tool.

Two reviewers (OM, KK) independently performed title and abstract screening and full-text review. Conflicts were resolved through discussion and consensus (OM, KK). Data were abstracted independently by one reviewer (OM) and verified for accuracy by a second reviewer (KK). A data abstraction form was created, with variables specified a priori and categorized into descriptive characteristics related to the study design, intervention, participants, and outcomes. The following study characteristics were systematically extracted: lead author, year, country, inpatient versus outpatient setting, single versus multicenter study, and study objective(s). Participant characteristics included population description, cancer type(s)/stage(s), number of participants, age, and sex. Psychosocial intervention characteristics included intervention description, therapeutic modality, delivery setting, mode of delivery, timing and duration, and intervention deliverer. Therapeutic modalities were categorized using the Fawzy (1999) theoretical model for designing psychosocial interventions, including education, behavioral training, psychotherapy, emotional support, and multimodal interventions.^18^ We performed a narrative synthesis of included studies to describe intervention characteristics and their impact on quality of life, anxiety, and depression.

### Risk of Bias Assessment

We performed a risk of bias assessment using the revised Cochrane risk of bias tool for randomized trials (RoB 2) tool (Version 22, August 2019) for all three outcomes.^19^ The risk of bias assessment was performed independently by one reviewer (OM).

## Results

### Search Results and Study Characteristics

**Fig. 1:**
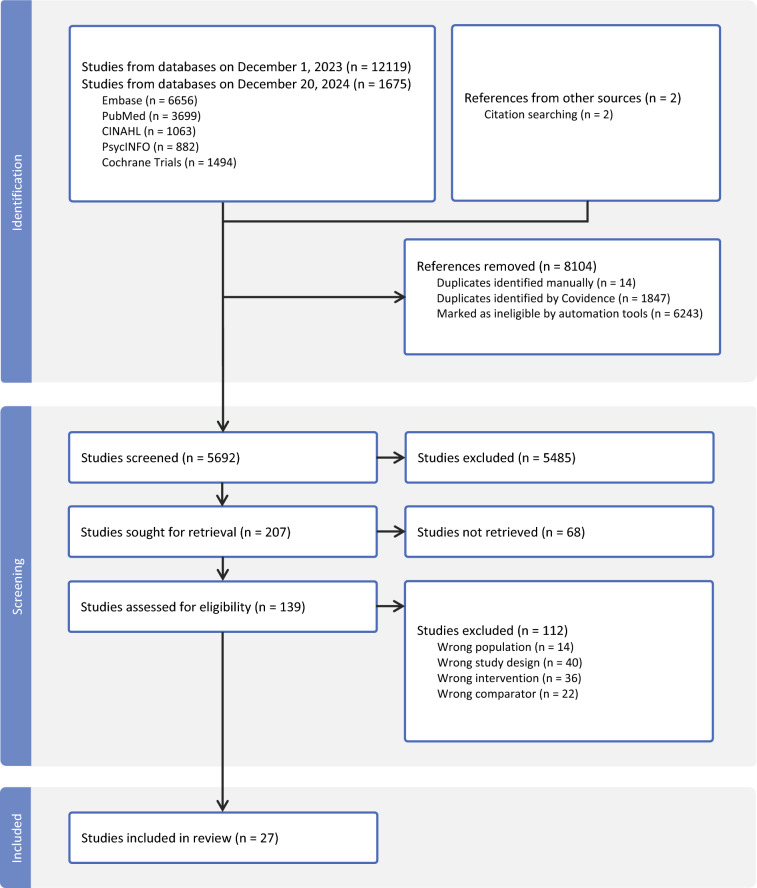
Preferred reporting items for systematic reviews and meta-analyses (PRISMA) flow diagram

We identified 12,119 studies on 1 December 2023, with an additional 1675 identified through the updated search in December 2024 (total: 13,794). The sources were PubMed (*n* = 3699), Embase (*n* = 6656), Cochrane Trials (*n* = 1494), CINAHL (*n* = 1063), and PsycInfo (*n* = 882). Additionally, we identified 2 studies through manual review. We removed 1861 duplicates using Covidence (*n* = 1847) and manually (*n* = 14), as well as 6243 studies that the Covidence RCT automation tool identified as ineligible. We performed title and abstract screening on 5692 studies and excluded 5485. Of the 207 identified for retrieval, 68 were inaccessible. Full-text review was conducted on the available 139 studies, of which 112 were excluded, leaving 27 studies that met our inclusion criteria. Figure 1 displays our PRISMA flow diagram.

The 27 included studies were published between 2005 and 2024 and conducted in Asia (*n* = 17, 63.0%), Europe (*n* = 8, 29.6%), and South America (*n* = 2, 7.4%). In Asia, most studies were conducted in China (*n* = 15, 88.2%), followed by Japan (*n* = 1, 5.9%) and Iran (*n* = 1, 5.9%). In Europe, studies were conducted in Denmark (*n* = 2, 25.0%), Germany (*n* = 2, 25.0%), Ireland (*n* = 1, 12.5%), Italy (*n* = 1, 12.5%), Spain (*n* = 1, 12.5%), and Poland (*n* = 1, 12.5%). In South America, both studies were conducted in Brazil. No included studies were conducted in North America.

All included studies were RCTs per the selection criteria, with 20 single-center (74.1%) and 5 multicenter (18.5%) trials. Most studies (*n* = 25, 92.6%) assessed the effect of one psychosocial intervention, while two studies (*n* = 2, 7.4%) assessed two interventions. A total of 16 studies (59.3%) reported the impact of the intervention(s) on quality of life^20–35^ using the following instruments: European Organisation for Research and Treatment of Cancer Quality of Life Questionnaire-Core 30 (EORTC-QLQ-C30),^20–25,29–31,33,34^ including two supplementary modules for colon (EORTC QLQ-CR38)^20^ and gastric (EORTC QLQ-STO22) cancer;^21,28^ Gastrointestinal Quality of Life Index (GIQLI);^22^ 36-Item Short Form Survey (SF-36);^26^ European Quality of Life-5 Dimensions (EQ-5D);^34^ World Health Organization Quality of Life-BREF (WHOQOL-BREF);^32^ and Functional Assessment of Cancer Treatment – Colorectal (FACT-C) (Table 1).^35^ Anxiety was assessed in 16 studies (59.3%)^20,25–27,30–32,34,36–43^ using the Hospital Anxiety and Depression Scale (HADS; HADS-A for anxiety),^20,25,30–32,34,36,40^ Self-rating Anxiety Scale (SAS),^26,27,34,39,41^ Spielberger State-Trait Anxiety Inventory (STAI),^37,38,42,43^ and Visual Analog Scale (VAS).^38^ Finally, depression was assessed in 12 studies (44.4%)^20,25–27,30–32,34,36,39–41^ using the HADS (HADS-D for depression)^20,25,30–32,34,36,40^ and Self-rating Depression Scale (SDS) (Table 2).^26,27,34,39,41^

**Table 1:** Quality of life instruments

Instrument	Description
EORTC QLQ-C30 (European Organisation for Research and Treatment of Cancer Quality of Life Questionnaire-Core 30)^44^	The EORTC QLQ-C30 is a 30-item questionnaire developed by the European Organisation for Research and Treatment of Cancer to assess quality of life in patients with cancer. It includes a global health status/QoL scale, five functional subscales (physical, role, cognitive, emotional, and social), three symptom subscales (fatigue, pain, and nausea/vomiting), and several single items addressing additional symptoms (e.g., dyspnea, appetite loss, sleep disturbance, constipation, diarrhea) and financial impact. The global health scale uses a 7-point response scale, while other items use a 4-point scale. Scores are transformed into a 0–100 scale, with higher scores indicating better functioning or greater symptom burden, depending on the subscale.
EORTC Modules QLQ-CR38 (Quality of Life Questionnaire – Colorectal Cancer module; 38 items)^45^ QLQ-STO22 (Quality of Life Questionnaire – Stomach Cancer module; 22 items)^46^	The EORTC QLQ-CR38 and QLQ-STO22 are disease-specific modules used alongside the QLQ-C30 to assess quality of life in patients with colorectal and gastric cancer, respectively. These modules assess symptoms, side effects, and functional limitations relevant to each cancer type.
GIQLI (Gastrointestinal Quality of Life Index)^47^	The GIQLI is a 36-item questionnaire designed to assess gastrointestinal symptoms, physical and psychological well-being, social functioning, and disease-specific concerns. Each item is scored from 0 (least desirable) to 4 (most desirable), with total scores ranging from 0 to 144. Higher scores reflect better gastrointestinal quality of life.
SF-36 (36-Item Short Form Survey)^48^	The SF-36 is a widely used generic measure of health-related quality of life. It includes eight domains: physical functioning, role limitations (physical and emotional), energy/fatigue, emotional well-being, social functioning, pain, and general health. Items are scored and transformed into a 0–100 scale using the RAND scoring method, where higher scores indicate better health status. RAND’s method uses simple linear transformations and does not apply norm-based scoring.
EQ-5D (European Quality of Life-5 Dimensions)^49^	The EQ-5D is a brief, standardized instrument that assesses five dimensions of health: mobility, self-care, usual activities, pain/discomfort, and anxiety/depression. Each dimension is rated at one of three (EQ-5D-3L) or five (EQ-5D-5L) levels of severity. The descriptive responses are converted to a single summary index score, typically anchored between 0 (death) and 1 (perfect health), though negative scores are possible. A visual analogue scale (VAS) from 0 to 100 is also included.
WHOQOL-BREF (World Health Organization Quality of Life – BREF; abbreviated version)^50^	The WHOQOL-BREF is a 26-item questionnaire developed by the World Health Organization to measure quality of life across four domains: physical, psychological, social, and environmental health. Items 1 and 2 assess overall quality of life and satisfaction with health. Higher scores indicate better self-perceived quality of life.
FACT-C (Functional Assessment of Cancer Therapy–Colorectal)^51^	The FACT-C is a 35-item questionnaire that measures quality of life in patients with colorectal cancer. It includes five subscales: physical, social, emotional, and functional well-being, plus a colorectal cancer-specific subscale. Items are rated on a 5-point Likert scale (0–4), and total scores range from 0 to 136, with higher scores reflecting better quality of life. The Trial Outcome Index (TOI), a clinically relevant summary score, combines physical, functional, and CRC-specific subscales.

EORTC QLQ-C30 European organisation for research and treatment of cancer quality of life questionnaire-core 30, QoL Quality of life, QLQ-CR38 Quality of life questionnaire—colorectal cancer module, QLQ-STO22 Quality of life questionnaire—stomach cancer module, GIQLI Gastrointestinal quality of life index, RAND Research and development corporation, EQ-5D European quality of life-5 dimensions, VAS Visual analogue scale, WHOQOL-BREF World health organization quality of life—BREF (abbreviated version), FACT-C Functional assessment of cancer therapy–colorectal, TOI Trial outcome index, CRC Colorectal cancer

**Table 2:** Anxiety and depression instruments

Instrument	Description
HADS (Hospital Anxiety and Depression Scale)	The HADS is a 14-item screening tool developed by Zigmond and Snaith (1983) to detect anxiety and depression in nonpsychiatric medical patients. It contains two subscales: HADS-A for anxiety and HADS-D for depression, each with seven items rated on a 4-point scale (0–3). Subscale scores range from 0 to 21. Scores of 0–7 are considered normal, 8–10 suggest mild symptoms, 11–14 indicate moderate symptoms, and 15–21 reflect severe anxiety or depression. Higher scores denote greater symptom severity.
STAI (State-Trait Anxiety Inventory)^52–54^	The STAI is a 40-item self-report instrument developed by Spielberger et al. (1970) to assess two types of anxiety: state anxiety (STAI-S) and trait anxiety (STAI-T). Each subscale consists of 20 items rated on a 4-point Likert scale (1 = not at all to 4 = very much so). Subscale scores range from 20 to 80. Higher scores indicate greater anxiety. While clinical cutoffs vary, scores of 20–30 are often considered low, 31–49 moderate, and 50 or above high anxiety.
STAI-SF (State-Trait Anxiety Inventory Short Form)^55^	The STAI-SF is a 6-item short form of the STAI-State scale developed by Marteau and Bekker (1992) to provide a rapid measure of current anxiety. It uses the same 4-point Likert scale as the full version. Scores are typically prorated to match the original 20-item scale, with higher scores indicating greater state anxiety.
VAS (Visual Analog Scale for Anxiety)^56^	The VAS is a simple, subjective measure of anxiety where individuals mark a point on a horizontal 10 cm line anchored by descriptors such as “no anxiety” (left end) and “worst possible anxiety” (right end). The score is the distance in millimeters from the left end, yielding a range from 0 to 100. Higher scores reflect greater anxiety severity.
SAS (Self-Rating Anxiety Scale)^57^	The SAS, developed by Zung (1971), is a 20-item self-report questionnaire used to assess the severity of anxiety symptoms. Items are rated on a 4-point scale (1 = a little of the time to 4 = most of the time). The raw score is summed and multiplied by 1.25 to yield a standardized score. A standardized score of ≥ 50 suggests the presence of clinically significant anxiety.
SDS (Self-Rating Depression Scale)^58^	The SDS, also developed by Zung (1965), mirrors the SAS but assesses depressive symptoms. It consists of 20 items rated on a 4-point scale, and the total raw score is multiplied by 1.25 to produce a standardized score. A score of ≥ 53 indicates clinically relevant depression, with higher scores representing greater symptom severity.

HADS Hospital anxiety and depression scale, STAI State-trait anxiety inventory, STAI-SF State-trait anxiety inventory short form, VAS Visual analog scale for anxiety, SAS Self-rating anxiety scale, SDS Self-rating depression scale

The number of participants randomized ranged from 34 to 336 participants. The most common cancer type was colorectal (*n* = 10, 37.0%), followed by gastric (*n* = 7, 25.9%), esophageal (*n* = 5, 18.5%), and liver (*n* = 3, 11.1%), with two studies (7.4%) comprising a combination of patients with esophageal, gastric, colon, and rectal cancer. In approximately half of the included studies (*n* = 14, 51.9%), a psychiatric disorder was an exclusion criterion.

### Psychosocial Intervention Characteristics of Included Studies

Psychosocial interventions were variable in terms of therapeutic modality and included education (*n* = 4, 14.8%), behavioral training (*n* = 4, 14.8%), psychotherapy (*n* = 4, 14.8%), emotional support (*n* = 2, 7.4%), bimodal with education and emotional support (*n* = 9, 33.3%), or multimodal including different combinations of therapeutic modalities (*n* = 4, 14.8%) (Table 3). Interventions also varied by timing relative to surgery. Six interventions (22.2%) were delivered exclusively in the preoperative period, most commonly as brief educational interventions administered in the days preceding surgery. A total of 11 interventions (40.7%) were initiated postoperatively, often after discharge, and typically involved ongoing education, emotional support, psychotherapy, or multimodal follow-up throughout the recovery period. Nine interventions (33.3%) spanned the perioperative period, beginning before surgery and continuing into the postoperative setting, while only one study (3.7%) did not clearly report intervention timing (Table 3).

**Table 3:** Intervention characteristics

Author, year	Modality	Delivery setting	Individual versus group	Mode of delivery	Timing	Duration
Haase 2005	Behavioral training	Inpatient, outpatient	Individual	Audio-visual	2 days preop to 1 week postop	~9 days
Ross 2005	Multimodal (education and emotional support)	Outpatient	Individual	In-person, telephone	2–3 months to 24 months postop	~22 months
Zhang 2013	Multimodal (other)	Inpatient, outpatient	Individual	In-person, printed materials, audio-visual	1 week pre-op to 2 weeks postop	~3 weeks
O’Connor 2014	Education	Outpatient	Individual	Printed materials, in-person	Preop meeting with stoma nurse	Not reported
Davoodi 2015	Multimodal (education and emotional support)	Inpatient, outpatient	Individual	In-person, printed materials, telephone	Discharge to 2 weeks after discharge	~2 weeks
Koplin 2016	Behavioral training	Inpatient, outpatient	Individual	Audio-visual	2 days preop to 30 days postop	~32 days
Qin 2017	Multimodal (education and emotional support)	Inpatient	Individual	In-person	Not reported	Not reported
Scarpa 2017	Psychotherapy	Inpatient	Individual	In-person	Admission to discharge	Hospital stay
Garcia 2018	Emotional support	Inpatient	Individual	In-person	1 day preop	30 min
Shao 2019	Education	Outpatient	Individual	Audio-visual	1 day preop	20 min
Wang 2019	Multimodal (education and emotional support)	Outpatient	Individual and group	In-person, printed materials, telephone	Postop (when patient deemed stable) to 12 months postop	~12 months
Baoyindeligeer 2020	Multimodal (education and emotional support)	Inpatient	Individual	In-person	Preop (not specified) to discharge	Not reported
Fang 2020	Multimodal (education and emotional support)	Inpatient, outpatient	Individual	In-person	Preop appointment to discharge	Not reported
Gao 2020	Multimodal (other)	Inpatient, outpatient	Individual and group	In-person, printed materials	Postop (when patient deemed stable) to 2 weeks after discharge	~2 weeks
Lin 2020	Psychotherapy	Outpatient	Group	In-person	7 days postop to 10 weeks after program start	10 weeks
Liu 2021	Education	Not reported	Individual	Audio-visual	1 day preop	20 min
Oliveira 2021	Emotional support	Outpatient	Individual	Telephone	5 days to 9 months after discharge	~9 months
Zhang 2021	Psychotherapy	Outpatient	Group	In-person	Within 1 month to 12 months after discharge	~12 months
Li 2022	Psychotherapy	Outpatient	Group	In-person	Within 1 month to 12 months after discharge	~12 months
Rocamora 2022	Behavioral training	Outpatient	Individual	Digital/online	Preop (not specified)	Not reported
Xu 2022	Multimodal (education and emotional support)	Inpatient, outpatient	Individual	In-person	Preop to postop (not specified)	Not reported
Yu 2022	Multimodal (education and emotional support)	Outpatient	Individual and group	Telephone, digital/online	Discharge to 6 months after discharge	~6 months
Bin 2023	Multimodal (other)	Not reported	Individual	In-person	Preop to postop (not specified)	Not reported
Hovdenak 2023	Multimodal (education and emotional support)	Outpatient	Individual and group	In-person, printed materials, digital/online, telephone	Within 45 days to 3 years postop	~3 years
Kasai 2023	Education	Not reported	Individual	Audio-visual, in-person	Preop (not specified)	Not reported
Liu 2023	Multimodal (other)	Outpatient	Individual and group	In-person, digital/online	0 to 6 months postop	~6 months
Wang 2024	Behavioral training	Inpatient	Group	In-person	4 days preop to 5 days postop	~9 days

#### Education

All four interventions that used education as the sole therapeutic modality were performed preoperatively.^36,38,40,42^ The content varied, but all studies included some combination of education about disease and treatment characteristics; the surgical procedure; surgical side effects and complications; recommendations for nutrition, sleep, and physical activity; and psychosocial support. In O’Connor et al., the intervention group received a tailored informational pamphlet and a nurse-led “guided tour” of the content.^36^ The study assessed changes in anxiety (HADS-A) and depression (HADS-D) before the intervention (before surgery) and after the intervention (after surgery but before hospital discharge, and 6 months after discharge). The intervention group had a significantly lower mean anxiety score than the control group (means not reported, *p* = 0.04) 6 months after discharge.^36^

In two studies, the educational intervention was a 20-min video delivered the day before surgery.^38,40^ Shao et al. measured anxiety (STAI and VAS) before the intervention (presurgery) and after the intervention (1 h before and 24 h after surgery).^38^ Intervention participants exhibited significantly lower mean anxiety scores postsurgery as measured by the STAI (1 h before: 47.87 ± 9.37 versus 54.14 ± 8.43, *p* < 0.001; 24 h after: 40.05 ± 8.81 versus 46.20 ± 8.66, *p* < 0.001) and VAS (1 h before: 5.29 ± 1.11 versus 5.75 ± 0.98, *p* = 0.013; 24 h after: 4.14 ± 0.86 versus 4.63 ± 0.78, *p* = 0.001).^38^ Liu et al. (2021) measured anxiety (HADS-A) and depression (HADS-D) before the intervention (before surgery) and after the intervention (1 h before and 24 h after surgery). Similarly, the intervention group scored significantly lower on anxiety (1 h before: 8.28 ± 1.76 versus 9.09 ± 2.08, *p* = 0.018; 24 h after: 7.28 ± 1.76 versus 8.05 ± 2.07, *p* = 0.026) and depression (1 h before: 7.11 ± 1.84 versus 8.31 ± 2.08, *p* = 0.001; 24 h after: 5.95 ± 1.62 versus 7.08 ± 1.90, *p* < 0.001) at study completion compared with the control group.^40^

Finally, the intervention evaluated in Kasai et al. included a 20-min video with additional presurgery counseling by surgeons.^42^ The authors measured anxiety (short form of the STAI) after the intervention (before surgery) and reported no significant difference in the median anxiety scores between the intervention and control groups.^42^

#### Behavioral Training

Four studies evaluated behavioral training interventions.^22,32,43,59^ Two interventions included mindfulness exercises.^32,43^ Rocamora et al. evaluated the effectiveness of an application-based intervention that provided mindfulness exercises during the preoperative period.^32^ The authors measured quality of life (WHOQOL-BREF), anxiety (HADS-A), and depression (HADS-D) before the intervention (before surgery) and after the intervention (at and 1 month after discharge), and found no significant differences in mean quality of life, anxiety, or depression between the intervention and control groups.^32^ The intervention described by Wang et al. (2024) included daily mindfulness meditation exercises beginning 4 days before surgery until 5 days after surgery in the inpatient setting.^43^ The authors measured anxiety (STAI) before the intervention (on admission), during the intervention (1 day before and after surgery), and after the intervention (5 days after surgery) and reported a significantly lower mean STAI score in the intervention group 1 day before and 1 day after surgery compared with the control group (1 day before surgery: 36.69 ± 7.04 versus 45.03 ± 9.12, *p* = 0.001; 1 day after surgery: 37.13 ± 12.02 versus 42.10 ± 8.29, *p* = 0.037).^43^

Two studies evaluated guided imagery and progressive muscle relaxation (PMR) versus standard of care.^22,59^ In both studies, intervention participants received a 12-min guided imagery or PMR tape with a portable audio player and were instructed to listen to the recording three times a day before and after surgery. Koplin et al. measured quality of life before the intervention (before surgery), during the intervention (3 and 7 days after surgery), and after the intervention (30 days after surgery) using the EORTC-QLQ-C30 and GIQLI.(22) Compared with the control group, participants in both the guided imagery and relaxation groups scored significantly lower on the cognitive functional subscale on postoperative day 3 (means not reported) and role functional subscale on postoperative day 7 (guided imagery versus control: 30.0 ± 41.0 versus 69.4 ± 38.9, *p* = 0.004; relaxation versus control: 28.6 ± 37.3 versus 69.4 ± 38.9, *p* = 0.007). Further, participants in the relaxation group scored significantly lower than those in the control group on the physical functional subscale on postoperative day 7 (41.9 ± 26.8 versus 67.8 ± 29.2, *p* = 0.006). In terms of the symptom subscales and items, participants in the guided imagery group scored significantly higher than those in the control group on the fatigue symptom subscale (80.2 ± 22.4 versus 60.4 ± 26.3, *p* = 0.03) and appetite loss item on postoperative day 3 (77.8 ± 34.3 versus 45.8 ± 38.2, *p* = 0.02), appetite loss item on postoperative day 7 (57.9 ± 34.9 versus 31.3 ± 33.3, *p* = 0.03), and dyspnea item on postoperative day 30 (42.1 ± 33.0 versus 15.6 ± 27.8, *p* = 0.02).^22^ The authors also reported a significantly lower mean quality of life (GIQLI score) in the guided imagery group compared with the control group on postoperative day 3 (85.7 versus 99.0, *p* < 0.04).^22^

#### Psychotherapy

Four studies evaluated psychotherapy interventions,^24,30,31,39^ of which two studies evaluated the effect of cognitive therapy interventions.^24,39^ The intervention outlined by Scarpa et al. consisted of psychological counseling, whereby a psychologist performed four in-person psychotherapy sessions with patients throughout their hospital admission, using a cognitive-interaction model that is the basis for cognitive behavioral therapy (CBT).^24^ The authors measured the impact of the intervention on quality of life using specific subscales and items of the EORTC-QLQ-C30 (global health status scale, fatigue symptom subscale, sleep disturbance item) before the intervention (before surgery) and after the intervention (after surgery at time of hospital discharge), and found that participants in the psychological counseling intervention group had 77% lower odds of experiencing quality of life impairment (difference of ≥ 10 points from admission to discharge) compared with the those in the control group after the intervention (OR 0.23, 95% CI 0.0–0.61, *p* = 0.003), with no significant differences noted in the fatigue symptom subscale or sleep disturbance item.^24^ Lin et al. also assessed the effectiveness of a cognitive therapy intervention, which used a 10-week stress management and psychological resilience training course on the basis of attention and interpretation therapy (AIT).^39^ The course included intensive teaching, transcendental meditation, emotional control training, mindfulness-based interventions, and acceptance and commitment therapy. It was delivered in-person in a group format postoperatively, starting 7 days after surgery and continuing for 10 weeks. The authors evaluated anxiety (SAS) and depression (SDS) before the intervention (after surgery) and after the intervention (10 weeks after enrollment) and found lower mean SAS (44.42 ± 3.64 versus 56.55 ± 4.21, *p* < 0.000) and SDS (43.95 ± 4.14 versus 55.12 ± 4.35, *p* < 0.000) scores post-study in the AIT group compared with the control.^39^

Both Zhang et al. (2021) and Li et al. evaluated a reminiscence therapy intervention, a therapeutic technique guiding individuals to process life experiences.^30,31^ In both studies, participants received group therapy sessions once or twice per month postoperatively, beginning 1 month after discharge and continuing for 12 months. Both studies measured quality of life (EORTC-QLQ-C30), anxiety (HADS-A), and depression (HADS-D) before the intervention (after surgery), during the intervention (at 3, 6, and 9 months), and after the intervention (at 12 months).^30^ Zhang et al. (2021) found that intervention group participants scored higher on the mean global health status scale at 12 months (means not reported, *p* = 0.032) and lower on the symptom scale at 6 months (means not reported, *p* = 0.048) compared with the control group.^30^ Similarly, Li et al. reported higher mean global health status in the reminiscence therapy group at 6 (71.3 ± 12.8 versus 66.3 ± 12.9, *p* = 0.048) and 12 (74.5 ± 12.9 versus 68.2 ± 13.3, *p* = 0.014) months compared with the control group.^31^ In terms of anxiety and depression scores, Zhang et al. (2021) reported a significantly lower mean anxiety score in the reminiscence therapy group at 6 (means not reported, *p* = 0.022), 9 (means not reported, *p* = 0.008), and 12 (means not reported, *p* = 0.004) months compared with the control group, with no significant differences in mean depression scores.^30^ Li et al. reported lower mean anxiety and depression scores in the intervention group compared with the control group at 9 (HADS-A: 6.8 ± 2.3 versus 7.8 ± 2.4, *p* = 0.039; HADS-D: 6.6 ± 2.0 versus 7.5 ± 2.2, *p* = 0.025) and 12 (HADS-A: 6.6 ± 2.4 versus 7.8 ± 2.6, *p* = 0.013; HADS-D: 6.3 ± 2.3 versus 7.7 ± 2.6, *p* = 0.005) months.^31^

#### Emotional Support

Two studies evaluated emotional support interventions.^29,37^ In Garcia et al., an in-person therapeutic listening session was delivered by a research team member in the inpatient setting 1 day before surgery.^37^ This 30-min session was designed to encourage participants to discuss their experience with hospitalization for cancer treatment. The authors measured anxiety (STAI) before and after the intervention (before surgery) and found no differences between the groups.^37^ In Oliveira et al., participants received outpatient monitoring by a nurse who provided postoperative guidance and support, including telephone follow-ups (at 5 days, 2 months, 4 months, and 9 months after hospital discharge).^29^ The authors measured quality of life (EORTC-QLQ-C30) before the intervention (before surgery), during the intervention (20 days, 3 months, and 6 months after surgery) and after the intervention (12 months after surgery) and found no differences in the global health status, functional, or symptom scales or subscales.^29^

#### Bimodal Interventions (Education and Emotional Support)

Nine studies evaluated interventions that combined education and support as therapeutic modalities.^20,21,23,25–27,33,35,60^ Three studies evaluated interventions involving upfront education, with follow-up support provided postoperatively.^21,25,35^ In Davoodi et al., an educational session occurred on the day of discharge and participants received a telephone follow-up from a research team member 2 weeks after discharge.^21^ The authors assessed quality of life (EORTC-QLQ-C30 and EORTC-QLQ-STO22) before the intervention (after surgery) and after the intervention (1 month after surgery), and found no between-group differences.^21^ In the intervention outlined by Hovdenak et al., an educational session occurred in the outpatient setting (within 45 days of surgery), followed by specialist nurse support by telephone and/or email for up to 3 years postsurgery.^35^ The authors assessed health-related quality of life (FACT-C) before the intervention (2 weeks after surgery) and after the intervention (3 years after surgery), reporting no statistically significant differences between groups.^35^ The intervention assessed by Wang et al. (2019) consisted of initial educational materials, followed by postoperative monthly in-person sessions, recreational activities, and telephone follow-up by a nurse. The intervention began when patients were deemed stable after surgery and continued for 12 months.^25^ The authors measured quality of life (EORTC-QLQ-C30), anxiety (HADS-A), and depression (HADS-D) before the intervention (after surgery), during the intervention (3, 6, and 9 months after surgery), and after the intervention (12 months after surgery).^25^ They reported significantly higher mean global health status (means not reported, *p* < 0.05) and functional scores (means not reported, *p* < 0.05) in the intervention group at 12 months; and significantly lower mean anxiety scores at 9 and 12 months (means not reported, *p* < 0.05) and depression scores at 12 months (means not reported, < 0.05).^25^

Two studies evaluated nurse-led outpatient follow-up programs that included education.^20,33^ Ross et al. assessed the effectiveness of an intervention involving ten home visits performed by a nurse or doctor with additional support offered via telephone, beginning 2–3 months after discharge and continuing for 24 months.^20^ Quality of life (EORTC-QLQ-C30 and EORTC-QLQ-CR38), anxiety (HADS-A), and depression (HADS-D) were measured during the intervention (3, 6, and 12 months after discharge) and after the intervention (24 months after discharge) with no reported differences between the intervention and control groups.^20^ Similarly, in Yu et al., participants were enrolled in a 6-month outpatient follow-up program beginning at the time of hospital discharge. Participants in this study were called by a nurse periodically and invited to join an online group with nursing support throughout the follow-up period. The authors measured quality of life (EORTC-QLQ-C30) at intervention completion (6 months after discharge) and found that intervention group participants scored significantly higher on all functional subscales and mean global health status, and scored lower on all symptom subscales and individual items compared with the standard of care group (all *p* < 0.05).^33^

Four studies evaluated nursing interventions grounded in psychological principles, using a combination of education and emotional support to promote well-being.^23,26,27,60^ In all interventions, participants received routine nursing and psychological nursing support.^23^ Qin et al. measured quality of life (EORTC-QLQ-C30) pre- and post-intervention, reporting significantly higher post-intervention scores on the physical (89.42 ± 12.35 versus 80.37 ± 14.32, *p* = 0.001), cognitive (84.10 ± 12.45 versus 76.54 ± 12.31, *p* = 0.0029), role (76.89 ± 10.65 versus 71.55 ± 13.14, *p* = 0.0277), and emotional (90.72 ± 8.27 versus 75.60 ± 10.01, *p* = 0.000) functional subscales in the intervention group, with no between-group differences in social function.^23^ Fang et al. measured anxiety (SAS) and depression (SDS) before the intervention (before surgery) and after the intervention (after surgery at hospital discharge), finding lower mean anxiety (48.76 ± 2.01 versus 50.10 ± 2.25, *p* = 0.001) and depression (39.68 ± 2.12 versus 40.92 ± 2.38, *p* = 0.003) scores posttreatment in the intervention group.^27^ Finally, Baoyindeligeer et al. assessed quality of life (SF-36), anxiety (SAS) and depression (SDS) before the intervention (before surgery) and after the intervention (14 days after surgery), reporting a significantly higher mean SF-36 score (means not reported, *p* < 0.05), and lower mean SAS (17.95 ± 3.02 versus 31.58 ± 3.39, *p* < 0.001) and SDS (19.44 ± 4.04 versus 33.62 ± 4.65, *p* < 0.001) scores 14 days postsurgery in the intervention group compared with the control group.^26^

#### Multimodal Interventions (Other Combinations)

Four studies evaluated psychosocial interventions consisting of > 2 therapeutic modalities.^28,34,41,61^ Two interventions included education, behavioral training, and emotional support.^34,61^ In the intervention outlined in Zhang et al. (2013), participants received 1-h in-person sessions with a therapist every other day, beginning 1 week before surgery and continuing 2 weeks after surgery. Sessions involved building trusting relationships, active listening, health education, psychological support, and behavioural training. Additionally, patients and families received peer-support in the early postoperative period.^61^ Liu et al. developed an offline-to-online cognitive behavioral stress management intervention, which occurred postoperatively and lasted up to 6 months. The offline component was delivered in the first 10 weeks after surgery and included weekly in-person group sessions containing didactic information and relaxation training. This was followed by the online component, where participants were invited to participate in cognitive behavioral stress management activities through an online platform. The authors evaluated quality of life (EORTC-QLQ-30 and EQ-5D), anxiety (HADS-A and SAS), and depression (HADS-D and SDS) before the intervention (after surgery), during the intervention (1 and 3 months after surgery), and after the intervention (6 months after surgery).^34^ The authors found that mean EQ-5D scores were significantly higher in intervention participants compared with the standard of care group at 1 (means not reported, *p* = 0.025) and 3 (means not reported, *p* = 0.030) months.^34^ The EORTC-QLQ-C30 mean global health status and functional scores were also significantly higher in the intervention group at 3 (means not reported, global health status: *p* = 0.027; functional score:* p* = 0.005) and 6 (means not reported, global health status: *p* = 0.001; functional score: *p* = 0.001) months.^34^ The EORTC-QLQ-C30 symptom score was significantly lower in the intervention group compared with the standard of care group at 3 months (means not reported, *p* = 0.014).^34^ Anxiety scores were significantly lower in the intervention group at 3 (means not reported, HADS-A: *p* = 0.004; SAS: *p* = 0.011) and 6 (means not reported, HADS-A: *p* = 0.008; SAS: 0.013) months.^34^ Depression scores were also significantly lower in the intervention group at 1 (means not reported, HADS-D: *p* = 0.020), 3 (means not reported, HADS-D: *p* = 0.009; SDS: *p* = 0.007), and 6 (means not reported, HADS-D: 0.007; SDS: *p* = 0.004) months.^34^

Two studies assessed interventions that included education, psychotherapy, and emotional support.^28,41^ In Bin et al., the intervention included in-person sessions with nurses, rehabilitation therapists, and psychological counselors, beginning preoperatively and extending postoperatively (timing not reported). The authors measured anxiety (SAS) and depression (SDS) before and after the intervention.^41^ After follow-up, the mean SAS and SDS scores were lower in the intervention group (SAS: 46.53 ± 2.30 versus 55.63 ± 2.35, *p* = 0.000; SDS: 47.01 ± 2.43 versus 56.21 ± 2.40, *p* = 0.000) compared with the control group.^41^ Gao et al. evaluated an intervention consisting of health education, behavior and lifestyle guidance, communication with rehabilitation experts, and psychological counseling in the inpatient setting after surgery, followed by telephone follow-up in the outpatient setting after discharge.^28^ The intervention was delivered by two nurses, beginning when patients were deemed stable until 2 weeks after hospital discharge. Quality of life (EORTC-QLQ-STO22) was assessed before the intervention (1 month before surgery) and after the intervention (1 month after surgery).^28^ One month after surgery, intervention group participants had a lower mean QLQ-STO22 score (54.26 ± 7.03 versus 60.17 ± 7.28, *p* < 0.001), reflecting fewer symptoms compared with the control group, such as lower mean scores for dysphagia (34.86 ± 2.17 versus 36.33 ± 3.10, *p* = 0.016), pain and discomfort (38.66 ± 3.08 versus 44.29 ± 3.72, *p* < 0.001), reflux symptoms (32.26 ± 2.76 versus 33.64 ± 3.10, *p* = 0.039), and emotional issues (49.55 ± 4.20 versus 53.56 ± 3.25, *p* < 0.001).^28^

### Risk of Bias Assessment

For the main outcome (quality of life), most studies (*n* = 11, 68.8%) had an overall high risk of bias according to RoB 2 scoring. Four studies (25%) had some concerns and only 1 study (6.3%) was found to have a low risk of bias. The RoB 2 assessments of the secondary outcomes (anxiety and depression) were similar. For anxiety, 12 (75%) had high risk of bias, 3 (18.8%) had some concerns, and 1 study (6.3%) had overall low risk of bias. For depression, risk of bias was high in eight studies (66.7%), moderate in three studies (25%), and low in one study (8.3%). These assessments are outlined in Table A2, Supplementary Material.

## Discussion

Our analysis included 27 studies published between 2005 and 2024 and conducted in Asia, Europe, and South America. These studies included adult patients diagnosed with GI cancer, primarily colorectal, gastric, esophageal, and liver cancer, who were awaiting elective surgery. The most common psychosocial interventions were bimodal approaches combining education and emotional support, followed by standalone and multimodal approaches.

Research suggests that psychosocial interventions offer benefits to patients with cancer, including increased disease understanding;^62,63^ reduced psychological and physical symptoms;^11^ improved quality of life, anxiety, depression, emotional well-being, and coping abilities;^11,62–66^ increased care satisfaction;^64,65^ and greater adherence to conventional treatments.^64^ This systematic review is the first to evaluate psychosocial interventions specifically in surgical patients with GI cancer. Consistent with broader oncology literature,^11,67–70^ our findings indicate that these interventions may be effective in this population, with 9 of 16 studies (56.3%) demonstrating significant improvements in quality of life;^23–26,28,30,31,33,34^ 12 of 16 studies (75%) reporting significant reductions in anxiety;^25–27,30,31,34,36,38–41,43^ and 8 of 12 studies (66.7%) reporting significant reductions in depression scores.^25–27,31,34,39–41^ However, substantial heterogeneity in intervention modalities, delivery settings, timing, and duration limited our ability to determine the most effective components and precluded meaningful comparisons by intervention characteristics, such as timing.

From an implementation perspective, the interventions included in this review likely differed substantially in feasibility. Implementation research emphasizes that clinical effectiveness alone is insufficient to guide intervention uptake in clinical practice and highlights the importance of evaluating implementation outcomes, such as acceptability, adoption, appropriateness, feasibility, and fidelity, in addition to effectiveness outcomes.^71–73^ Frameworks such as the Consolidated Framework for Implementation Research (CFIR)^74^ and the Reach, Effectiveness, Adoption, Implementation, and Maintenance (RE-AIM) framework^75^ provide useful approaches for understanding the factors that influence implementation and real-world integration of interventions. In the present review, these implementation-related outcomes were rarely reported, which limited our ability to determine which interventions may be most practical and scalable within surgical oncology settings. From a clinical perspective, the findings outlined in this study support the integration of psychosocial support into surgical oncology care for patients with GI cancer, particularly during periods of high perioperative distress in an attempt to enhance quality of life and psychosocial health. Although no single intervention component emerged as clearly superior, lower resource-intensive approaches such as structured education, telephone-based follow-up, video-based preparation, and digital support may represent more pragmatic entry points for implementation in clinical practice because they are less resource-intensive and potentially easier to deliver within existing clinical pathways compared with interventions involving repeated in-person sessions, multidisciplinary teams, or long-term follow-up. Future trials would benefit from incorporating implementation science principles and hybrid effectiveness–implementation study designs to evaluate both clinical and implementation outcomes and better support the implementation, scale-up, and sustainability of psychosocial interventions.

Psychosocial factors are increasingly recognized for influencing postoperative outcomes.^5,76^ A prospective observational study conducted in 2019 revealed the association between preoperative psychosocial risk factors and 30-day complications among elective GI cancer surgery patients, where medically comorbid patients with multiple psychosocial risk factors had greater than three times the odds of experiencing a postoperative complication compared with patients with one or no risk factors.^5^ Interestingly, approximately half of the studies in the present review excluded patients with psychosocial risk factors (e.g., diagnosed psychiatric illnesses), a population likely to benefit from psychosocial support. This is a significant limitation, as these patients could gain the most from psychosocial interventions. Future studies should aim to include and stratify patients by the presence of psychosocial risk factors to identify subgroups who derive the greatest benefit from these interventions.

Most studies exhibited a high risk of bias. This aligns with prior reviews, such as Newell et al., who identified 34 studies assessing the effectiveness of psychological interventions for patients with cancer broadly and found that despite an increase in RCTs over time, the methodologic quality and rigor of interventional trials was suboptimal.^77^ Similarly, Steel et al. identified eight studies evaluating psychosocial interventions for patients with GI cancer and reported variable study quality, but noted improvements in reporting and methodological rigor over time.^11^ The high risk of bias in the published literature in this field has important implications for how these findings should be interpreted. In the context of the current review, this reduces confidence in the observed improvements in quality of life, anxiety, and depression and suggests that the magnitude of benefit may be overestimated. Future RCTs evaluating psychosocial interventions in surgical patients should adhere to minimal reporting standards, such as the Consolidated Standards of Reporting Trials (CONSORT) guidelines, to improve quality and strengthen the conclusions and recommendations that can be drawn. Furthermore, the development and adoption of core outcomes for psychosocial intervention research would improve comparability and facilitate high-quality meta-analyses to generate more robust evidence to support implementation of psychosocial interventions within surgical practice.

This systematic review has several limitations. As with any systematic review, relevant studies may not have been identified and included in our sample, and therefore, unintentionally excluded. Of the articles identified for full-text review, 68 were inaccessible and therefore could not be evaluated for inclusion. This may have limited the comprehensiveness of the review and could have introduced bias if the inaccessible studies differed systematically from those that were accessible, for example by region, publication source, or indexing. Additionally, significant heterogeneity among the included studies in terms of study populations and psychosocial interventions, and the relatively small number of studies within each therapeutic modality category, limited our ability to compare the effectiveness of different therapeutic modalities and approaches. Furthermore, this review focused on outcomes related to quality of life, anxiety, and depression. Standardizing outcome measures would have improved comparability and allowed for pooled analyses.

## Conclusions

This systematic review provides evidence that psychosocial interventions may benefit patients with GI cancer undergoing surgery, particularly in improving quality of life and reducing anxiety and depression. However, the methodological limitations of existing studies underscore the need for more robust research in this field. Future studies should prioritize adhering to reporting standards and including patients with known psychosocial risk factors. Addressing these gaps will help generate stronger evidence to guide surgical practice in meeting the psychosocial needs of this population.

## Supplementary Information

Below is the link to the electronic supplementary material.

Supplementary file1 (DOCX 24 kb)
