# Supplementary material for: Psychosocial Interventions for Patients Undergoing Gastrointestinal Cancer Surgery: A Systematic Review
Source: Ann Surg Oncol. 2026 May 25;33(8):7358–72. doi: 10.1245/s10434-026-19788-7 (PMC13337738; doi:10.1245/s10434-026-19788-7)
Supplement: Supplementary file 1 — Supplementary file1 (DOCX 24 kb) [file 10434_2026_19788_MOESM1_ESM.docx]

**Supplementary Material**

**Table A1.** Search strategy.

| **Database** | **Search strategy** |
| --- | --- |
| Medline (OVID) | 1. exp "Neoplasms"/ 2. (neoplas* or cancer* or tumor* or tumour* or malign* or oncolog* or carcinoma*).tw,kf. 3. 1 or 2 4. exp "Digestive System Surgical Procedures"/ or exp "Colorectal Surgery"/ 5. (esophagectom* or gastrectom* or pyloromyotom* or cholecystectom* or enterectom* or colectom* or hemicolectom* or proctocolectom* or hepatectom* or pancreatectom* or proctectom* or "liver transplant*").tw,kf. 6. (exp "Surgical Procedures, Operative"/ or exp "General Surgery"/ or exp "Surgical Oncology"/) and exp "Digestive System"/ 7. ((esopha* or gastr* or stomach* or gallbladder* or intestine* or bowel* or colon* or colorectal* or liver or pancreatic or "bile duct*" or "retro peritonal sarcoma*" or "retroperitonal sarcoma*" or pancrea* or hepat* or biliar* or rectal* or rectum* or anus or anal) and (surg* or operat* or resect* or excision* or removal* or cytoreduct* or perioperative* or preoperative* or postoperative*)).tw,kf. 8. or/4-7 9. exp "Psychosocial Intervention"/ or exp "Mental Health Services"/ or exp "Internet-Based Intervention"/ 10. ((educ* or psycho* or social* or behavioral or behavioural) and intervention*).tw,kf. 11. ((cognitive or therap*) adj3 (behavioral or behavioural)).tw,kf. 12. (psychosocial* or "psycho social*" or psychological or mindful* or CBT or counselling or counseling).tw,kf. 13. or/9-12 14. 3 and 8 and 13 15. limit 14 to english language |
| Embase | 1. 'neoplasm'/exp 2. (neoplas* OR cancer* OR tumor* OR tumour* OR malign* OR oncolog* OR carcinoma*):ab,ti,kw 3. #1 OR #2 4. 'abdominal surgery'/exp OR 'esophagectomy'/exp 5. (gastrectom* OR pyloromyotom* OR cholecystectom* OR enterectom* OR colectom* OR hemicolectom* OR proctocolectom* OR hepatectom* OR pancreatectom* OR proctectom* OR esophagectom* OR "liver transplant*"):ab,ti,kw 6. (('surgery'/exp OR 'surgical oncology'/exp) AND ('digestive system'/exp)) 7. ((abdominal OR abdomen OR gastr* OR stomach* OR gallbladder* OR intestine* OR colon* OR colorectal* OR bowel* OR liver OR pancreatic OR "bile duct*" OR "retro peritonal sarcoma*" OR "retroperitonal sarcoma*" OR pancrea* OR hepat* OR biliar* OR rectal* OR rectum* OR anus OR anal) and (surg* OR operat* OR resect* OR excision* OR removal* OR cytoreduct* OR perioperative* OR postoperative*)):ab,ti,kw 8. #4 OR #5 OR #6 OR #7 9. ‘psychosocial intervention’ OR ‘mental health service’/exp OR ‘web-based intervention’ 10. ((educ* OR psycho* OR social* OR behavioral OR behavioural) AND intervention*):ab,ti,kw 11. ((cognitive OR therap*) NEAR/3 (behavioral OR behavioural)):ab,ti,kw 12. (psychosocial* OR "psycho social*" OR psychological OR mindful* OR CBT OR counselling OR counseling):ab,ti,kw 13. #9 OR #10 OR #11 OR #12 14. #3 AND #8 AND #13 15. #14 AND [english]/lim |
| Cochrane Trials | 1. MeSH descriptor: [Neoplasms] explode all trees 2. (neoplas* or cancer* or tumor* or tumour* or malign* or oncolog* or carcinoma*) 3. #1 OR #2 4. MeSH descriptor: [Digestive System Surgical Procedures] explode all trees 5. MeSH descriptor: [Colorectal Surgery] explode all trees 6. (esophagectom* or gastrectom* or pyloromyotom* or cholecystectom* or enterectom* or colectom* or hemicolectom* or proctocolectom* or hepatectom* or pancreatectom* or proctectom* or (liver NEXT/1 transplant*)) 7. MeSH descriptor: [Surgical Procedures, Operative] explode all trees 8. MeSH descriptor: [General Surgery] explode all trees 9. MeSH descriptor: [Surgical Oncology] explode all trees 10. MeSH descriptor: [Digestive System] explode all trees 11. ((esopha* or gastr* or stomach* or gallbladder* or intestine* or bowel* or colon* or colorectal* or liver or pancreatic or "bile duct" or "bile ducts" or ("retro peritonal" NEXT/1 sarcoma*) or (retroperitonal NEXT sarcoma*) or pancrea* or hepat* or biliar* or rectal* or rectum* or anus or anal) and (surg* or operat* or resect* or excision* or removal* or cytoreduct* or perioperative* or preoperative* or postoperative*)) 12. (#7 OR #8 OR #9) AND (#10) 13. #4 OR #5 OR #6 OR #11 OR #12 14. MeSH descriptor: [Psychosocial Intervention] explode all trees 15. MeSH descriptor: [Mental Health Services] explode all trees 16. MeSH descriptor: [Internet-Based Intervention] explode all trees 17. ((educ* or psycho* or social* or behavioral or behavioural) and intervention*) 18. ((cognitive or therap*) NEAR/3 (behavioral or behavioural)) 19. (psychosocial* or (psycho NEXT/1 social*) or psychological or mindful* or CBT or counselling or counseling) 20. #14 OR #15 OR #16 OR #17 OR #18 OR #19 21. #3 AND #13 AND #20 in Trials |
| PsycInfo | 1. DE "Neoplasms" 2. (neoplas* OR cancer* OR tumor* OR tumour* OR malign* OR oncolog* OR carcinoma*) 3. S1 or S2 4. gastrectom* OR pyloromyotom* OR cholecystectom* OR enterectom* OR colectom* OR hemicolectom* OR proctocolectom* OR hepatectom* OR pancreatectom* OR proctectom* OR esophagectom* OR "liver transplant*") 5. (DE "Surgery" OR DE "Organ Transplantation" OR DE "Postsurgical Complications" OR DE "Transection" OR DE "Surgical Patients") AND (DE "Digestive System" OR DE "Gastrointestinal System" OR DE "Intestines" OR DE "Stomach" OR DE "Liver" OR DE "Gastrointestinal Disorders" OR DE "Colon Disorders) 6. ((abdominal OR abdomen OR gastr* OR stomach* OR gallbladder* OR intestine* OR colon* OR colorectal* OR bowel* OR liver OR pancreatic OR "bile duct*" OR "retro peritonal sarcoma*" OR "retroperitonal sarcoma*" OR pancrea* OR hepat* OR biliar* OR rectal* OR rectum* OR anus OR anal) and (surg* OR operat* OR resect* OR excision* OR removal* OR cytoreduct* OR perioperative* OR postoperative*)) 7. S4 or S5 or S6 8. DE “Psychosocial Interventions” OR DE “Mental Health Services” OR DE “Digital Interventions” 9. ((educ* OR psycho* OR social* OR behavioral OR behavioural) AND intervention*) 10. ((cognitive OR therap*) N3 (behavioral OR behavioural)) 11. (psychosocial* OR "psycho social*" OR psychological OR mindful* OR CBT OR counselling OR counseling) 12. S8 OR S9 OR S10 OR S11 13. S3 AND S7 AND S12 14. S13 AND English limit |
| Cinahl | 1. (MH "Neoplasms+") 2. TI ( (neoplas* OR cancer* OR tumor* OR tumour* OR malign* OR oncolog* OR carcinoma*) ) OR AB ( (neoplas* OR cancer* OR tumor* OR tumour* OR malign* OR oncolog* OR carcinoma*) ) 3. S2 OR S3 4. (MH "Surgery, Digestive System+") 5. TI(gastrectom* OR pyloromyotom* OR cholecystectom* OR enterectom* OR colectom* OR hemicolectom* OR proctocolectom* OR hepatectom* OR pancreatectom* OR proctectom* OR esophagectom* OR "liver transplant*") OR AB(gastrectom* OR pyloromyotom* OR cholecystectom* OR enterectom* OR colectom* OR hemicolectom* OR proctocolectom* OR hepatectom* OR pancreatectom* OR proctectom* OR esophagectom* OR "liver transplant*") 6. (MH "Surgery, Operative+") AND (MH "Digestive System+") 7. TI((abdominal OR abdomen OR gastr* OR stomach* OR gallbladder* OR intestine* OR colon* OR colorectal* OR bowel* OR liver OR pancreatic OR "bile duct*" OR "retro peritonal sarcoma*" OR "retroperitonal sarcoma*" OR pancrea* OR hepat* OR biliar* OR rectal* OR rectum* OR anus OR anal) AND (surg* OR operat* OR resect* OR excision* OR removal* OR cytoreduct* OR perioperative* OR postoperative*)) OR AB((abdominal OR abdomen OR gastr* OR stomach* OR gallbladder* OR intestine* OR colon* OR colorectal* OR bowel* OR liver OR pancreatic OR "bile duct*" OR "retro peritonal sarcoma*" OR "retroperitonal sarcoma*" OR pancrea* OR hepat* OR biliar* OR rectal* OR rectum* OR anus OR anal) AND (surg* OR operat* OR resect* OR excision* OR removal* OR cytoreduct* OR perioperative* OR postoperative*)) 8. S4 or S5 or S6 or S7 9. (MH “Psychosocial Intervention+”) OR (MH “Mental Health Services+”) OR (MH “Internet-Based Intervention+”) 10. TI ((educ* OR psycho* OR social* OR behavioral OR behavioural) AND intervention*) OR AB ((educ* OR psycho* OR social* OR behavioral OR behavioural) AND intervention*) 11. TI ((cognitive OR therap*) N3 (behavioral OR behavioural)) OR AB ((cognitive OR therap*) N3 (behavioral OR behavioural)) 12. TI (psychosocial* OR "psycho social*" OR psychological OR mindful* OR CBT OR counselling OR counseling) OR AB (psychosocial* OR "psycho social*" OR psychological OR mindful* OR CBT OR counselling OR counseling) 13. S9 OR S10 OR S11 OR S12 14. S3 AND S8 AND S13 15. S14 AND English limit |

**Table A2.** Risk of bias assessment based on quality of life, anxiety, and depression outcomes.

| **Author, year** | **Domain 1 –**  **Randomization process** | **Domain 2 –**  **Deviations from the intended interventions** | **Domain 3 – Missing outcome data** | **Domain 4 –**  **Measurement of the outcome** | **Domain 5 –**  **Selection of the reported result** | **Overall risk of bias** |
| --- | --- | --- | --- | --- | --- | --- |
| **Quality of life outcome** | | | | | | |
| Ross, 2005 | Low | Some concerns  Low | Low | Low | Low | Some concerns |
| Davoodi, 2015 | Low | Low  Low | Low | Some concerns | Low | Some concerns |
| Koplin, 2016 | Some concerns | Some concerns  Low | Low | Some concerns | Some concerns | High |
| Qin, 2017 | Some concerns | Low  Low | Low | Some concerns | High | High |
| Scarpa, 2017 | Low | Some concerns  Low | Low | Some concerns | Low | High |
| Wang, 2019 | Low | Low  Low | Low | Low | Low | Low |
| Baoyindeligeer, 2020 | Some concerns | Low  Low | Low | Some concerns | Low | High |
| Fang, 2020 | Some concerns | Low  Low | Low | High | Some concerns | High |
| Gao, 2020 | Some concerns | High  Low | Some concerns | High | Low | High |
| Oliveira, 2021 | Some concerns | Some concerns  Low | Low | Some concerns | Low | High |
| Zhang, 2021 | Low | Low  Low | Low | Some concerns | Low | Some concerns |
| Li, 2022 | Low | Low  Low | Low | Some concerns | Low | Some concerns |
| Rocamora, 2022 | Low | Some concerns  Low | Low | Some concerns | Low | High |
| Yu, 2022 | Some concerns | Some concerns  Low | Low | Low | Low | High |
| Hovdenak, 2023 | Low | Some concerns  Low | Low | Some concerns | Low | High |
| Liu, 2023 | Some concerns | Low  Low | High | Some concerns | Low | High |
| **Anxiety outcome** | | | | | | |
| Ross, 2005 | Low | Some concerns  Low | Low | Low | Low | Some concerns |
| O’Connor, 2014 | Low | Some concerns  Low | Low | High | Low | High |
| Garcia, 2018 | Low | Some concerns  Low | Low | Some concerns | Low | High |
| Shao, 2019 | Some concerns | Low  Low | Low | Some concerns | Low | High |
| Wang, 2019 | Low | Low  Low | Low | Low | Low | Low |
| Baoyindeligeer, 2020 | Some concerns | Low  Low | Low | Some concerns | Low | High |
| Fang, 2020 | Some concerns | Low  Low | Low | Some concerns | Low | High |
| Lin, 2020 | Some concerns | Low  Low | Low | Some concerns | Low | High |
| Liu, 2021 | Some concerns | Low  Low | Low | Some concerns | Low | High |
| Zhang, 2021 | Low | Low  Low | Low | Some concerns | Low | Some concerns |
| Li, 2022 | Low | Low  Low | Low | Some concerns | Low | Some concerns |
| Rocamora, 2022 | Low | Some concerns  Low | Low | Some concerns | Low | High |
| Bin, 2023 | Some concerns | Some concerns  Low | High | Some concerns | Low | High |
| Kasai, 2023 | Some concerns | Low  Low | Low | Some concerns | Low | High |
| Liu, 2023 | Some concerns | Low  Low | High | Some concerns | Low | High |
| Wang, 2024 | Some concerns | High  Low | Low | Some concerns | Low | High |
| **Depression outcome** | | | | | | |
| Ross, 2005 | Low | Some concerns  Low | Low | Low | Low | Some concerns |
| O’Connor, 2014 | Low | Some concerns  Low | Low | High | Low | High |
| Wang, 2019 | Low | Low  Low | Low | Low | Low | Low |
| Baoyindeligeer, 2020 | Some concerns | Low  Low | Low | Some concerns | Low | High |
| Fang, 2020 | Some concerns | Low  Low | Low | Some concerns | Low | High |
| Lin, 2020 | Some concerns | Low  Low | Low | Some concerns | Low | High |
| Liu, 2021 | Some concerns | Low  Low | Low | Some concerns | Low | High |
| Zhang, 2021 | Low | Low  Low | Low | Some concerns | Low | Some concerns |
| Li, 2022 | Low | Low  Low | Low | Some concerns | Low | Some concerns |
| Rocamora, 2022 | Low | Some concerns  Low | Low | Some concerns | Low | High |
| Bin, 2023 | Some concerns | Some concerns  Low | High | Some concerns | Low | High |
| Liu, 2023 | Some concerns | Low  Low | High | Some concerns | Low | High |
